# Molecular Dynamics Insights into TAS1R2 Transmembrane Domain Activation

**DOI:** 10.3390/ijms262311464

**Published:** 2025-11-26

**Authors:** Yongcheng Lu, Xinyi Ma, Ziyue Meng, Meng Cui

**Affiliations:** 1 Department of Pharmaceutical Sciences, School of Pharmacy and Pharmaceutical Sciences, Bouvé College of Health Sciences, Northeastern University, Boston, MA 02115, USA; 2 Center for Drug Discovery, Northeastern University, Boston, MA 02115, USA

**Keywords:** G-protein-coupled receptor (GPCR), sweet taste receptor, MD simulations, protein-ligand interactions, receptor activation, conformational changes

## Abstract

Sweet taste receptors (STRs) are class C G protein-coupled receptors (GPCRs) that function as heterodimers of TAS1R2 and TAS1R3. These receptors possess multiple binding sites and can be activated by a wide range of sweet-tasting compounds. Interestingly, TAS1R2 alone or even its extracellular domain-truncated form (TAS1R2-TMD), can act as a functional receptor. Previous studies demonstrated that the sweetener S819 and the sweet inhibitor amiloride act through the transmembrane domain (TMD) of TAS1R2; however, the molecular mechanisms underlying these ligand-specific effects remain unclear, largely due to the historical lack of experimentally determined full-length STR structures. Recent breakthroughs in cryo-EM structural determination of the full-length TAS1R2/TAS1R3 complex now offer an unprecedented opportunity to elucidate receptor activation mechanisms at atomic resolution. In this study, we investigated ligand-induced conformational dynamics of hTAS1R2-TMD using microsecond-scale molecular dynamics (MD) simulations on three systems: hTAS1R2-TMD/S819 (agonist-bound), hTAS1R2-TMD/amiloride (antagonist-bound), and hTAS1R2-TMD (apo). Comparative analyses revealed that agonist and antagonist binding distinctly modulate key structural switches, including the conserved ionic lock (E6.35-R3.50), which stabilizes the inactive state and disrupts upon activation. Notably, we identified a novel salt bridge (D7.32-R3.32) that forms preferentially in the active state, potentially serving as a unique molecular switch for TAS1R2. Additional analyses uncovered ligand-specific rearrangements in hydrogen-bonding and hydrophobic interaction networks. These results provide atomistic insights into how agonists and antagonists differentially modulate TAS1R2 activation and lay a structural foundation for designing novel sweeteners and taste modulators.

## 1. Introduction

The human sweet taste receptor (STR) is a heterodimer made of TAS1R2 (T1R2) and TAS1R3 (T1R3), which belong to the class C family of G protein-coupled receptors (GPCRs) [1,2]. This Class GPCRs share a modular structure with three main parts: a large extracellular Venus flytrap domain (VFTD), a cysteine-rich domain (CRD), and a seven-transmembrane domain (7TMD) that connects to the G protein and drives signal transduction [3]. Previous studies using sweet receptor chimeras, mutants, and molecular modeling have identified at least five potential binding sites in the heteromeric receptor [4,5,6,7,8,9]. Receptor activity induced by the artificial sweeteners aspartame and neotame implicate residues in the VFTD of human T1R2 [4,8,10,11,12,13], while natural sugars bind to the VFTDs of both T1R2 and T1R3 [9]. In contrast, the sweeteners cyclamate and neohesperidine dihydrochalcone (NHDC), and the sweet-taste inhibitor lactisole [4,6,7,8], act on the TMD of human T1R3, and the sweeteners perillartine [14] and S819 act on the TMD of human T1R2 [15]. Furthermore, receptor activity due to the sweet-tasting protein brazzein depends on the CRD of human T1R3 [5].

Recent cryo-electron microscopy (cryo-EM) studies have revealed full-length structures of the human sweet receptor in both apo and ligand-bound forms [16,17,18]. These structures show an asymmetric heterodimer in which sucralose binds only to the VFTD of TAS1R2, while the G protein α-subunit interacts mainly with the TAS1R2 TMD [16]. These results give valuable insight into the overall receptor organization and how ligand binding may trigger activation.

Experimental findings support the idea that the TAS1R2-TMD can function independently. Chimeric receptor experiments showed that amiloride, a sweet-taste inhibitor, acts through direct interaction with the TAS1R2 heptahelical domain rather than TAS1R3, involving residues in TM3, TM5, and TM7 [19,20]. This inhibition varies across species, suggesting that specific TMD residues play critical roles in allosteric modulation [19]. In contrast, several compounds also activate the TAS1R2-TMD directly. Perillartine, a natural sweetener, can activate monomeric TAS1R2 even in the absence of TAS1R3, indicating that the TAS1R2 alone has ligand-binding and signaling ability. Moreover, the synthetic agonist S819 binds to the TMD of TAS1R2 and enhances receptor activation [14,15,21]. Altogether, these studies indicate that TAS1R2 is not just a passive structural subunit but can actively shift between inactive and active states in response to ligand binding.

While previous modeling studies have characterized activation mechanisms in the full TAS1R2/TAS1R3 heterodimer, including TM6 rearrangements and key ligand-binding residues [22] and dimer interface transitions [23], the intrinsic activation properties of the isolated TAS1R2 TMD remain unexplored. How agonists and antagonists differentially modulate TAS1R2-specific molecular switches, and whether TAS1R2 possesses unique activation mechanisms distinct from the heterodimer context, remain open questions. Addressing this gap requires a detailed structural and dynamic understanding of the TAS1R2 TMD alone, which forms the focus of the present study.

In this work, we used microsecond-scale molecular dynamics (MD) simulations of a truncated TAS1R2 TMD to study how the agonist S819 and the antagonist amiloride induce different activation patterns. By comparing agonist-bound and antagonist-bound states, we analyzed how ligand binding affects key structural switches such as ionic lock (E6.35-R3.50), a conserved motif that stabilizes the inactive conformation and breaks during activation [24,25]. We also identified a new salt bridge, D7.32-R3.32, that forms preferentially in the active state and may serve as a unique molecular switch for TAS1R2. In addition, our analysis revealed ligand-specific rearrangements in hydrogen-bond and hydrophobic networks. These findings provide an atom-level view of how agonists and antagonists differently modulate TAS1R2 activation and offer new clues for the rational design of sweeteners and taste modulators.

## 2. Results

### 2.1. Agonist-Induced Ionic Lock Broken in T1R2 TMD During 1 µs MD Simulations

**Figure 1:**
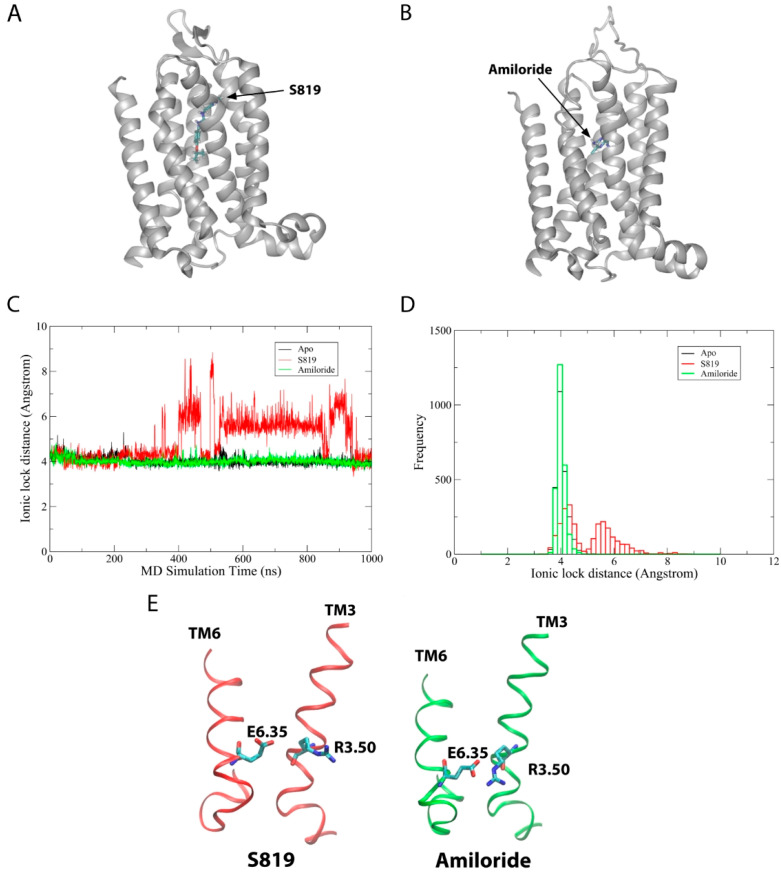
MD simulations on T1R2-TMD/Apo, T1R2-TMD/S819 and T1R2-TMD/Amiloride systems for 1 μs. (**A**) T1R2-TMD/S819 complex structure. (**B**) T1R2-TMD/Amiloride complex structure. (**C**) Ionic lock distances of the receptors as a function of simulation time (ns). (**D**) Histogram of D. (**E**) Representative snapshots showing ion lock broken in T1R2-TMD/S819 and formed in T1R2-TMD/Amiloride.

To understand ligand-induced activation mechanism in the T1R2 TMD, we performed 1 µs MD simulations on T1R2-TMD/APO, T1R2-TMD/Amiloride (antagonist-bound), and T1R2-TMD/S819 (agonist-bound) states for three replicas each. The structures of the T1R2-TMD/Amiloride and T1R2-TMD/S819 complexes (Figure 1A,B) were predicted by molecular docking using Induced Fit docking (IFD) tool of Schrodinger (2024-3) software [26]. Figures S1 and S2 show the root-mean-square deviation (RMSD) and radius of gyration (Rg) of the T1R2-TMD during MD simulations across all systems and replicas. Figure 1C,D shows the ionic lock distances (R3.50-E6.35) as a function of simulation time (see Figure S3 for replicas). The ionic locks for the T1R2-TMD/APO and T1R2-TMD/Amiloride systems were formed around 4 Å and were stable throughout the 1 µs simulations (Video S1). In contrast, the ionic lock for the T1R2-TMD/S819 system was broken (ranged from 4 to 8.5 Å) for the majority of the simulation time (Video S2). During the 1 µs MD simulations, the maximum ionic lock distance for the T1R2-TMD/S819 was approximately 8.5 Å, comparable to the experimental active state structure of beta2 adrenergic receptor, which is 9.97 Å (PDBID: 6KR8) [27]. Figure 1E shows representative snapshots of the ionic lock between R3.50 and E6.30 in the T1R2-TMD/Amiloride (ionic lock formed) and T1R2-TMD/S819 (ionic lock broken) systems from MD simulations. These findings suggest that agonist binding induces allosteric effects leading to ionic lock disruption. To further explore this mechanism, we analyzed ligand-receptor contact patterns during the simulations.

**Figure 2:**
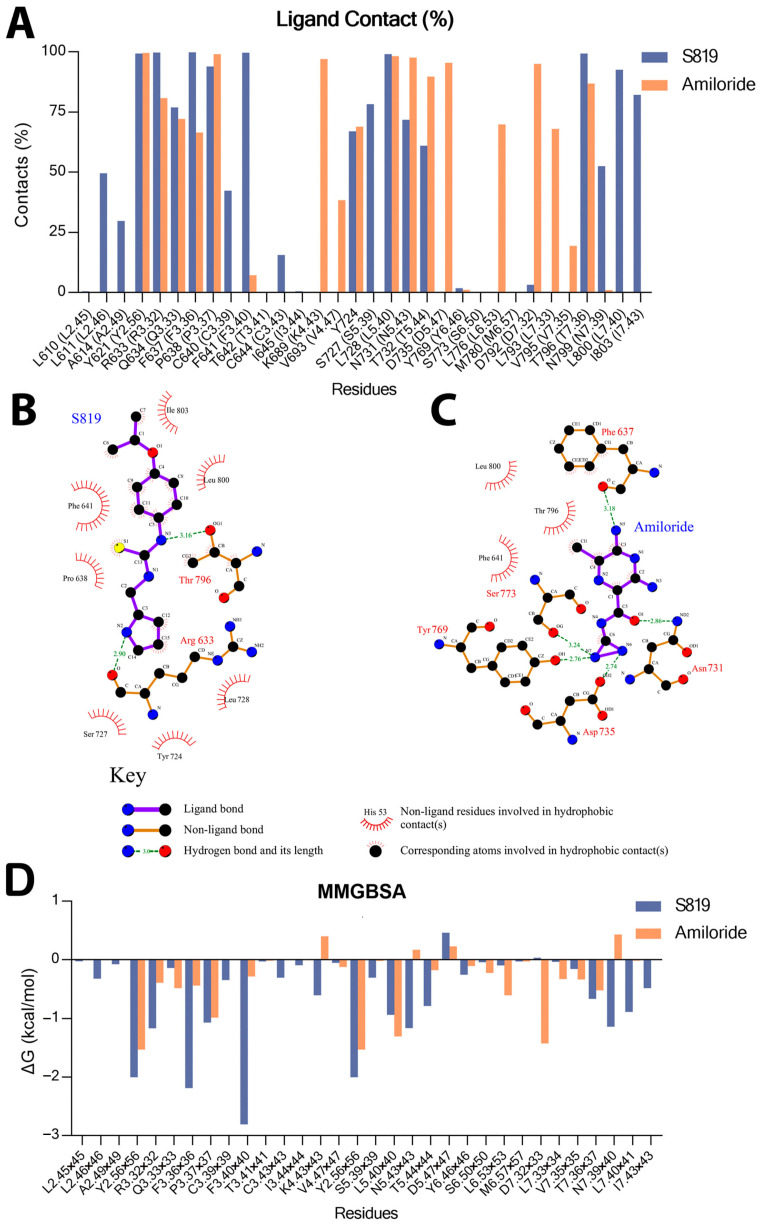
The binding site residues in the T1R2 receptor interact with S819 and Amiloride. (**A**) Percentage contacts of the binding site residues in T1R2 interactions with S819 and Amiloride during MD simulations (100–1000 ns). (**B**,**C**) 2D ligand−receptor interaction plots for S819 and Amiloride, respectively. Both snapshots were taken from the last frames of the simulations (at 1 µs). (**D**) Per-residue MM/GBSA decomposition of binding free energy for S819 and Amiloride, the energetic contributions of the contact residues are shown in (**A**).

Figure 2A presents the percentage of contacts between T1R2 binding-site residues and the compounds S819 and amiloride during the 1 µs MD simulations, while Figure 2D shows the corresponding per-residue MM/GBSA energy decomposition for these interacting residues.

S819 maintained frequent and stable interactions mainly with residues in TM3, TM5, and the upper region of TM7. The most prominent contacts included F3.40 (100% occupancy, ΔG = −2.81 kcal·mol^−1^) and L7.40 (99.5%, ΔG = −0.89 kcal·mol^−1^)**,** which likely contribute to ligand stabilization within the pocket. These interactions appear to promote a more flexible arrangement of TM3 and TM7, consistent with the conformational tendencies observed in the agonist-bound state.

In comparison, amiloride exhibited a stronger preference for residues located in the lower region of TM7, particularly D7.32 (99.7%, ΔG = −1.43 kcal·mol^−1^), with limited contact to TM3 residues such as F3.40 (9.6%, ΔG = −0.29 kcal·mol^−1^). Although amiloride also interacted with D5.47, its overall contact pattern suggests stabilization of a more compact binding pocket, consistent with an inactive-like conformation. Figure 2B,C illustrate the differences in binding patterns between the two lig-ands by Ligplot program [28].

A possible explanation for the disruption of the ionic lock was found by examining how S819 binds to nearby residues close to this region. F3.40, which is adjacent to R3.50 on TM3, stayed in contact with S819 during the entire simulation (100% occupancy), but did not interact with amiloride. This constant interaction likely perturbs the local helical environment, destabilizing the R3.50-E6.35 interaction and promoting ionic lock dissociation.

### 2.2. Agonist-Induced Conformation Changes in T1R2

**Figure 3:**
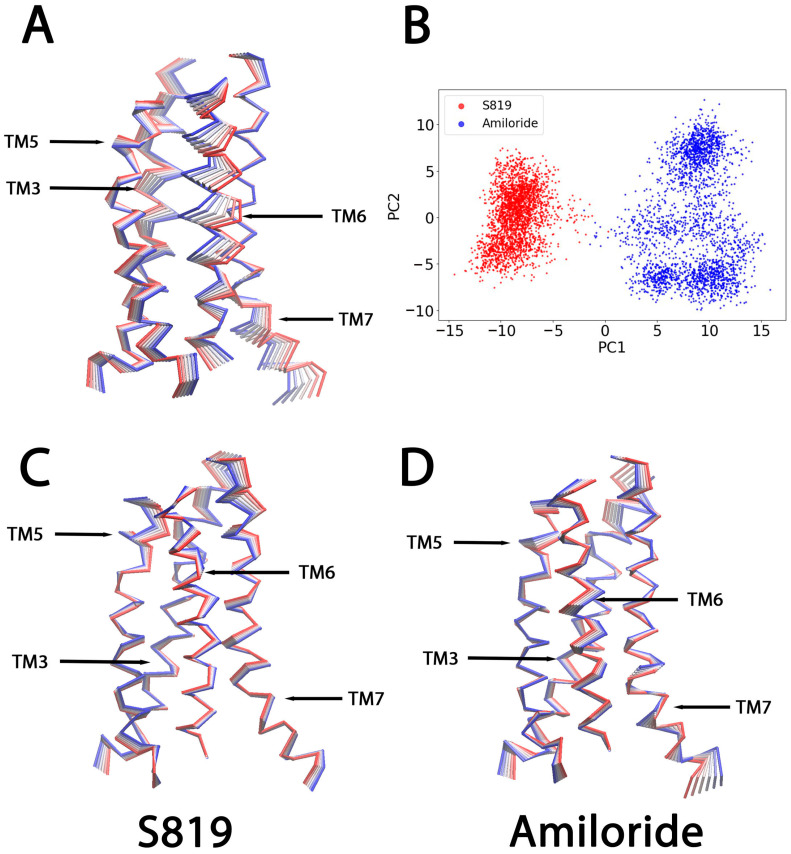
(**A**) The first eigenvectors (EVs) from combined Principal Component Analysis (PCA) of T1R2-TMD/S819 and T1R2-TMD/Amiloride based on the MD simulations (100–1000 ns; Cα atoms of the receptor). The TM3, 5, 6, 7 of the T1R2 receptor structures were shown as Cα traces (six frames colored from blue to red). (**B**) Projection of the PCA results onto the first two principal components (PC1 and PC2) in 2D space. Red points represent the S819 system, and blue points represent the Amiloride system. (**C**) The corresponding analysis for the S819-bound T1R2-TMD, showing the main conformational transition along PC1. (**D**) The corresponding analysis for the amiloride-bound T1R2-TMD, showing the main conformational transition along PC1.

In addition to showing that S819 binding leads to ionic lock disruption, we performed principal component analysis (PCA) on the combined T1R2-TMD/S819 and T1R2-TMD/Amiloride trajectories (100–1000 ns). PCA of the full protein showed a clear separation between the S819- and amiloride-bound ensembles in PC1-PC2 space (Figure S4), indicating that the two ligands stabilize distinct global conformations. Analysis of PC1 contributions revealed that extracellular loop 2 (ECL2) showed the largest displacements, suggesting a possible link between movements in the extracellular region and activation of the transmembrane domain. Additional PCA of the independent T1R2-TMD/S819 and T1R2-TMD/amiloride trajectories (100–1000 ns) is provided in Figure 3C,D.

To focus more specifically on activation-related changes, we performed a second PCA limited to TM3, TM5, TM6, and TM7 helices identified as important from contact and ionic lock analyses. The projection of their Cα atoms onto PC1 and PC2 again showed clear separation between the two ligand-bound states (Figure 3B). The S819-bound ensemble occupied a distinct area of conformational space, with little overlap with the amiloride-bound state. PC1, which captured the most variance, reflected the major differences between these two systems.

To identify which parts of the transmembrane domain contributed most to these changes, we visualized the first eigenvector (EV1) as displacement vectors mapped on the receptor structure (Figure 3A). Two areas showed strong signals. The first was the middle and extracellular parts of TM6, where large vectors indicated that flexibility is concentrated in the upper half of the helix. The second was the intracellular end of TM7, which includes glycine residues G7.46 and G7.49. These glycines increase backbone flexibility because they lack side chains, in line with the glycine-rich motif we identified in TM7.

**Figure 4:**
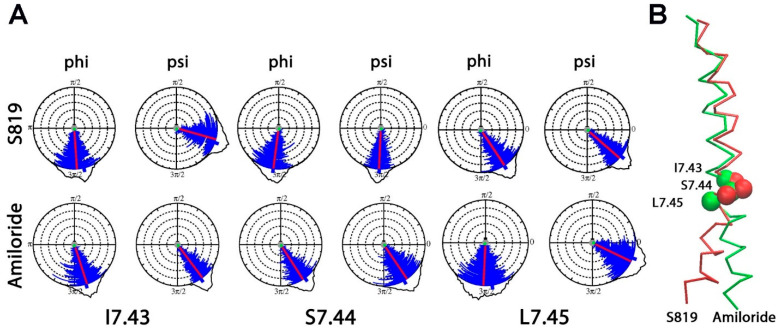
Agonist-induced conformational changes on the TM7 of T1R2. (**A**) Dial plots for phi/psi angle distributions of the residues I7.43, S7.44, and L7.45 in the TM7 for T1R2-TMD/S819 and T1R2-TMD/Amiloride (100–1000 ns). (**B**) TM7 conformation comparison between T1R2-TMD/S819 (red) and T1R2-TMD/Amiloride (green) during MD simulations based PCA results.

Backbone dihedral angle analysis supported these observations and showed clear changes between the two ligand conditions in the intracellular segment of TM7 (Figure 4A,B). For I7.43, the phi angle changed from −86.24° (S819 system) to −72.14° (Amiloride system), but the psi angle changed from −15.76° (S819 system) to −72.14° (Amiloride system). At S7.44, the phi angle moved from −97.38° (S819 system) to −55.81° (Amiloride system) and the psi angle from −92.48° (S819 system) to −55.81° (Amiloride system). For L7.45, the phi angle shifted from −56.45° (S819 system) to −92.73° (Amiloride system) and the psi angle from −41.08° (S819 system) to −25.73° (Amiloride system). These coordinated changes indicate substantial rearrangement of the TM7 intracellular backbone and are consistent with the large movements captured by EV1 in this region.

DSSP (Dictionary of Secondary Structure in Proteins) secondary structure analysis (Figure S5) showed π-helical character in the TM7 intracellular region in the amiloride condition. A π helix has a wider pitch than an α helix and imposes a more constrained geometry, which can stabilize the inactive state. In the S819 condition, this π-helical feature was reduced, and the segment adopted a more flexible α-helical arrangement. This transition from a constrained π helix in the inactive state to a more flexible helical structure in the active state provides the local adaptability needed for the TM7 intracellular segment to reorganize during activation.

### 2.3. Salt Bridge Interaction Network Changes in T1R2 by S819 Activation

**Figure 5:**
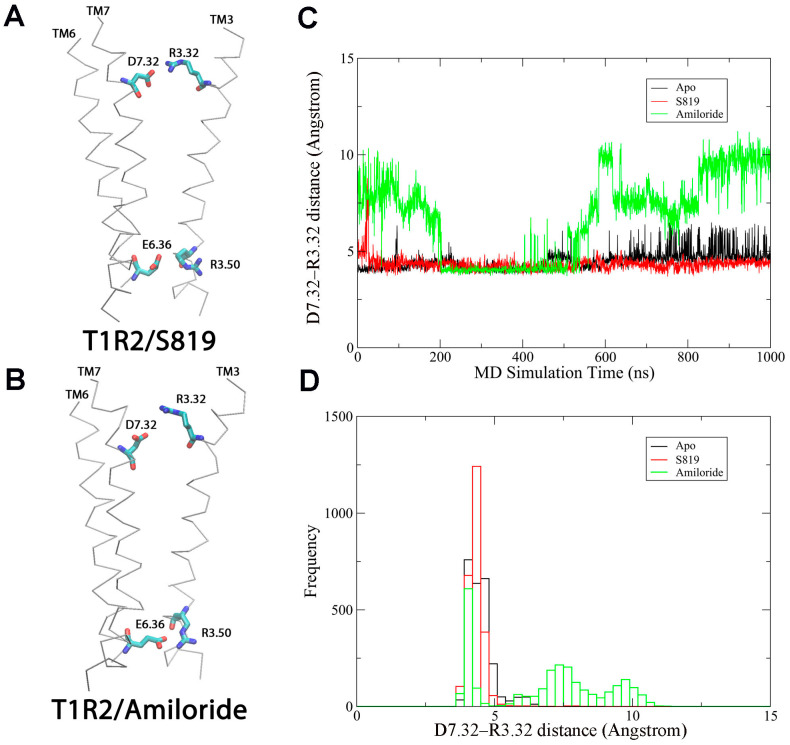
Comparison of key salt bridge interactions between T1R2-TMD/S819 and T1R2-TMD/Amiloride based on MD simulations (100–1000 ns). (**A**,**B**) The key salt bridge residues in T1R2-TMD/S819 and T1R2-TMD/Amiloride. (**C**) Salt bridge distances between residues D7.23 and R3.32. (**D**) Histograms of distributions for the distance between D7.23 and R3.32. E6.35-R3.50 is the ionic lock of T1R2.

Salt bridge interactions play a key role in maintaining receptor structure and function. In our 1 µs MD simulations, we observed the breakage of the ionic lock (E6.35-R3.50) in the T1R2-TMD/S819 system (Figure 1), a hallmark of GPCR activation. To explore other salt bridge interactions involved in this process, we systemically analyzed the receptor’s salt bridge interaction network and identified a second key interaction: the D7.32-R3.32 salt bridge, connecting TM7 and TM3. This interaction showed opposite behavior compared to the ionic lock during activation (Figure 5A,B and Figure S6).

In the amiloride-bound (inactive) state, the D7.32-R3.32 pair remained dissociated throughout the simulation, with inter-residue distances consistently above 10 Å (Figure 5C). In contrast, this salt bridge formed and stabilized in the S819-bound (active) state, maintaining distances under 4 Å for most of the trajectory. The frequency distribution of these distances is shown in Figure 5D.

This inverse relationship, breaking of the E6.35-R3.50 ionic lock and simultaneous formation of the D7.32-R3.32 salt bridge, might suggest a coordinated molecular switch. The mechanism behind this switching can be explained by ligand contact patterns. In the amiloride-bound state, D7.32 is heavily occupied (99.7% contact), preventing it from interacting with R3.32. In contrast, S819 interacts with D7.32 only 7.3% of the time, leaving the residue available to form the inter-helical salt bridge (Figure 2).

The D7.32-R3.32 salt bridge likely serves several roles in stabilizing the active conformation: (1) it supports the new TM3-M7 positioning following ionic lock breakage, (2) it helps anchor TM7 in its activated rotational state, and (3) it provides alternative stabilization at the TM3-TM6-TM7 interface to compensate for the loss of the E6.35-R3.50 constraint.

Thus, D7.32 functions as a molecular switch blocked by an antagonist in the inactive state, but free to engage in stabilizing interactions in the active state. This dual behavior may make D7.32 a key determinant of receptor activation and a potential target for pharmacological modulation.

It is worth noting that although the D7.23-R3.32 salt bridge is disrupted in the antagonist-bound state, it remains formed in both the agonist-bound and apo states. We interpret this as the antagonist interfering with salt-bridge formation, whereas the agonist promotes its stabilization. The apo state may exhibit some basal activity, consistent with the ionic-lock behavior observed in Figure S3. Figure 5 and Figure S6 show the D7.23-R3.32 salt-bridge distance, while Figure 1 and Figure S3 present the E6.35-R3.50 ionic-lock distance as a function of time over the 1 µs MD simulations.

### 2.4. Hydrogen Bond Interaction Network Changes in T1R2 by S819 Activation

**Figure 6:**
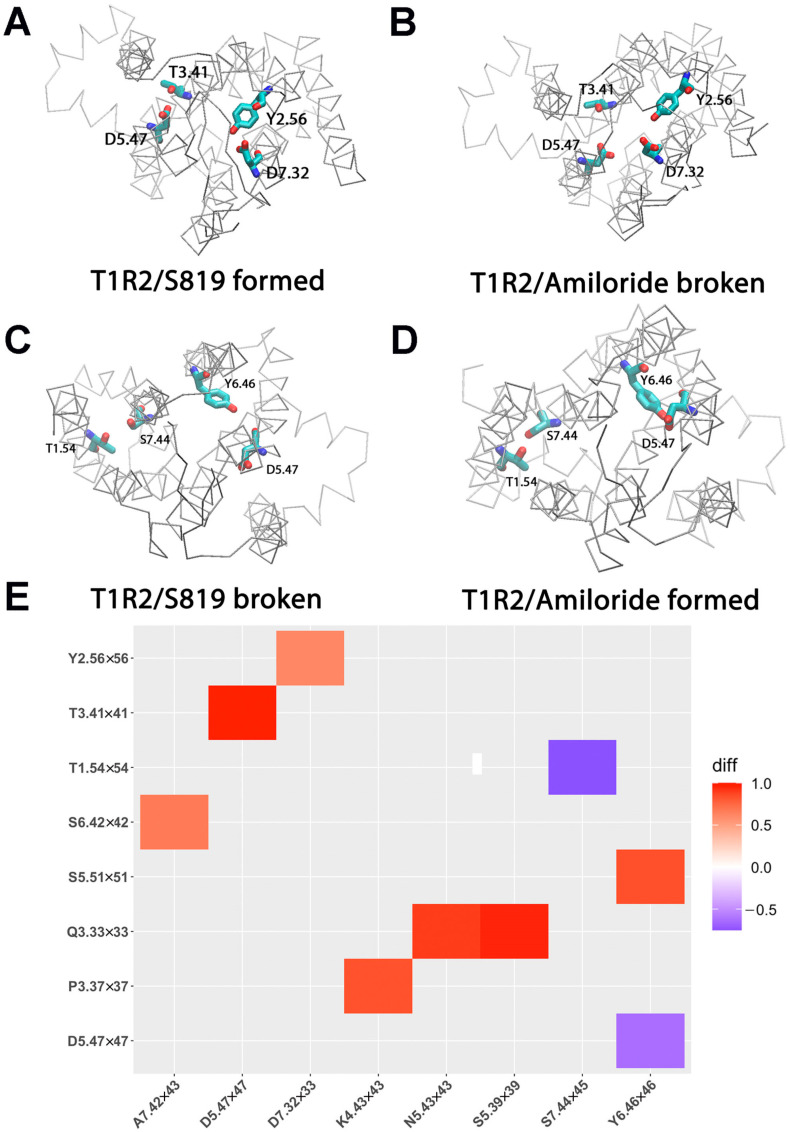
Comparison of hydrogen bond networks between T1R2-TMD/S819 and T1R2-TMD/Amiloride. (**A**) Hydrogen bond formed in T1R2-TMD/S819 system. (**B**) Hydrogen bond broken in T1R2-TMD/Amiloride system. (**C**) Hydrogen bond broken in T1R2-TMD/S819 system. (**D**) Hydrogen bond formed in T1R2-TMD/Amiloride system. (**E**) Heatmap plot of hydrogen bond pairs (red: hydrogen bond formed; blue: hydrogen bond disrupted).

Hydrogen bonds are essential for protein structure and function. They help maintain the fold of the receptor and support key secondary structures such as α helices and β sheets. To investigate their role in T1R2 activation, we carried out a systematic hydrogen bond network analysis based on MD simulation trajectories. Using the Simulaid program [29], we compared the differences in hydrogen bond occupancy between the T1R2-TMD/S819 and T1R2-TMD/Amiloride systems. The results are shown in a heatmap representation (Figure 6E), where red squares indicate an increase in hydrogen bond occupancy in the S819 system, while blue squares indicate a decrease compared to the amiloride system. The detailed hydrogen bond formation results are given in Table S1.

This analysis highlighted four hydrogen bonds that play a central role in distinguishing active and inactive states. The hydrogen bond between Y2.56 and D7.32 connected TM2 and TM7 in 82% of frames in the S819 system but only 8% of frames in the amiloride system (Figure 5A). This difference is explained by ligand occupancy: in the antagonist-bound state, amiloride maintained strong contact with D7.32 (99.7% frequency), preventing it from engaging in hydrogen bonding, whereas in the S819-bound state the residue was largely free (7.3% contact) to establish this stabilizing TM2-TM7 connection.

A second key interaction was observed between T3.41 and D5.47, linking TM3 and TM5. This bond formed in 79% of S819 frames but was almost absent in amiloride simulations (5%) (Figure 5B). Because T3.41 is located directly next to F3.40, which interacts continuously with S819, the presence of the agonist likely promotes formation of this TM3-TM5 bond, strengthening coupling between the two helices during the receptor activation.

By contrast, the hydrogen bond between Y6.46 and D5.47, which couples TM6 and TM5, was stable in the amiloride system (76% occupancy) but largely disrupted in the S819-bound state (12%) (Figure 5C). In the inactive receptor, this bond restricts TM6 movement, while its loss during S819 binding allows TM6 to undergo the conformational changes captured in PCA.

Finally, the hydrogen bond connecting T1.54 and S7.44 was maintained in 68% of amiloride frames but only 15% of S819 frames, indicating further reorganization of TM7 during the active state.

### 2.5. Hydrophobic Interaction Network Changes in T1R2 by S819 Activation

**Figure 7:**
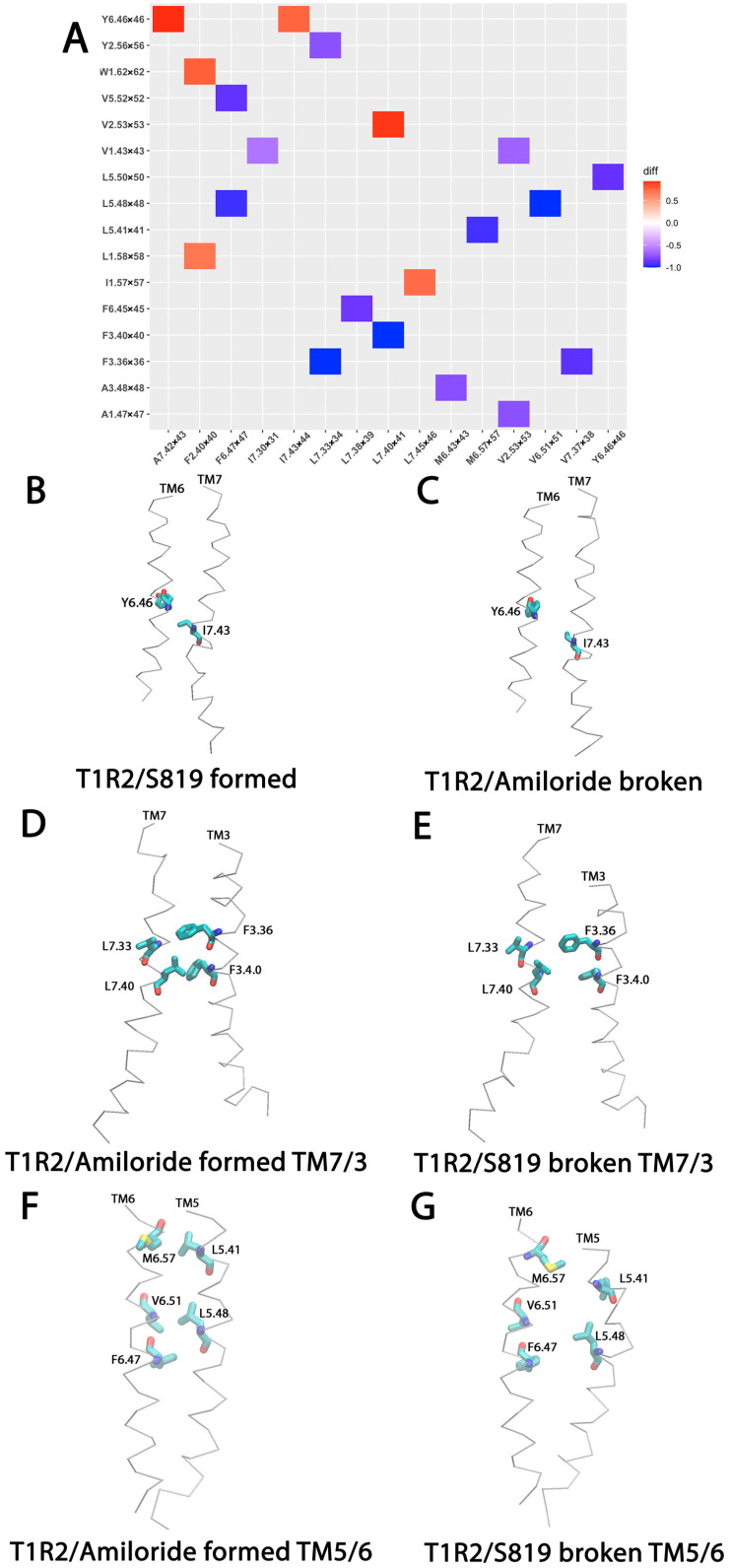
Hydrophobic interactions in T1R2-TMD/S819 and T1R2-TMD/Amiloride system. (**A**) Heatmap plot of hydrophobic interactions. (**B**) Hydrophobic interaction formed in the T1R2-TMD/S819 system. (**C**) Hydrophobic interaction formed in T1R2-TMD/Amiloride system. (**D**,**F**) Hydrophobic interaction formed in T1R2-TMD/Amiloride system, TM7/3 interaction (**D**) and TM5/6 interaction (**F**). (**E**,**G**) Hydrophobic interaction broken in T1R2-TMD/S819 system, TM7/3 interaction (**E**) and TM5/6 interaction (**G**).

To investigate the role of hydrophobic interactions in T1R2 activation, we analyzed hydrophobic contact networks using MD trajectories and compared the contact fractions between the T1R2-TMD/S819 and T1R2-TMD/Amiloride systems with the Simulaid program. A hydrophobic contact was defined as two non-polar residues within 5 Å. The results are presented as a heatmap (Figure 7A), where red indicates increased contacts in the S819 system and blue indicates decreased contacts compared to the amiloride system. The detailed hydrophobic interaction results are given in Table S2.

The heatmap revealed a major reorganization of the hydrophobic core during activation. Most hydrophobic contacts unique to the inactive state were disrupted in the presence of S819, while a new network of active-state interactions emerged. This shift demonstrates a remodeling of the transmembrane packing architecture. In the S819-bound, the most prominent changes were concentrated in the TM7-TM6-TM2 region (Figure 7B,C). Y6.46 strengthening its hydrophobic interactions with I7.43 on TM7. These contacts formed an interconnected hydrophobic cluster that stabilizes the active conformation and supports the TM6 rearrangements captured by PCA.

By contrast, hydrophobic interaction characteristics of the inactive state were largely lost upon activation. In the amiloride-bound, the TM7-TM3 interface was stabilized by close packing of L7.33 with F3.36, and L7.40 with F3.40. (Figure 7D,E). At the same time, TM6 and TM5 were coupled by a dense set of contacts, including V6.51 with L5.48, F6.47 with both L5.48, and M6.57 with L5.41 (Figure 7F,G). This hydrophobic packing held TM7 closely against TM3 and restricted TM6 movement by anchoring it to TM5. As a result, the transmembrane bundle was locked in the inactive conformation, preventing the structural rearrangements required for activation.

Unlike salt bridges and hydrogen bonds, which depend on electrostatic interactions between polar residues, hydrophobic interactions are driven by the tendency of non-polar residues to cluster together in aqueous environments. This hydrophobic effect plays a central role in stabilizing protein structure, especially in transmembrane domains where the lipid bilayer modulates these contacts.

### 2.6. E6.35A Mutation Disrupts the Inactive-State Ionic Lock

To further validate our computational predictions regarding the ionic lock and key ligand-contact residues, we conducted additional 1 μs MD simulations with point mutations in three critical residues: E6.35A, D7.32A, and F3.40A.

**Figure 8:**
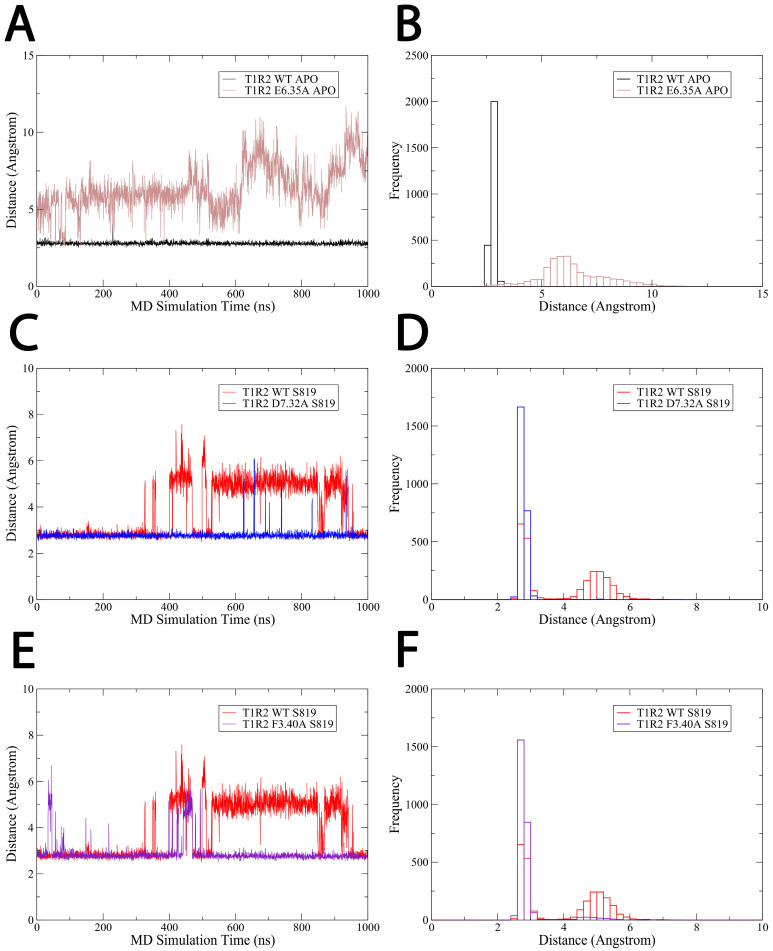
Time evolution and distributions of the ionic-lock distance (R3.50-E6.35) in wild-type and mutant systems. (**A**,**B**) Wild-type apo (black) versus E6.35A mutant (brown). (**C**,**D**) Wild-type S819-bound (red) versus D7.32A mutant (blue) (**E**,**F**) Wild-type S819-bound (blue) versus F3.40A mutant (purple). Time-series plots (**left**) and corresponding histograms (**right**) depict how mutations alter the stability of the ionic-lock interaction.

To assess whether E6.35 is essential for maintaining the ionic lock in the inactive state, we simulated the apo receptor carrying the E6.35A mutation. Distance measurements between A6.35 and R3.50 showed complete disruption of the ionic interaction (Figure 8A). While the wild-type apo receptor maintained a stable E6.35-R3.50 distance, consistent with a formed salt bridge, the E6.35A mutant exhibited broken salt bridge, This confirms that E6.35 is the obligate salt bridge partner in the ionic lock and that its removal alone is sufficient to disrupt this key inactive-state constraint, even without ligand binding.

### 2.7. D7.32A Mutation Prevents Agonist-Induced Ionic Lock Disruption

To further investigate the role of D7.32 in activation, particularly its dual function as an amiloride binding site in the inactive state and as a salt bridge partner for R3.32 in the active state, we simulated the S819-bound system with a D7.32A mutation. Remarkably, this mutation reversed the agonist’s typical effect. While the wild-type S819-bound system exhibited ionic lock disruption (E6.35-R3.50), the D7.32A mutant maintained a stable ionic lock distance (Figure 8C,D).

### 2.8. F3.40A Mutation Abolishes S819 Agonist Activity

To determine the functional significance of F3.40A, a residue showing the largest difference in contact frequency between S819 (100%) and amiloride (9.6%), we simulated the S819-bound receptor with an F3.40A mutation. This mutation completely abolished S819’s ability to break the ionic lock (Figure 8E,F).

## 3. Discussion

The ionic lock, formed between conserved arginine and acidic residues, is a well-known molecular switch in GPCR activation. In Class A GPCRs, the R3.50-E/D6.30 interaction holds the receptor in the inactive state, and its break is needed for activation and G protein coupling [30]. Our MD simulations show that this mechanism is also present in T1R2, even though it belongs to Class C GPCRs, which usually have different structural features. We observed that agonist S819 breaks the R3.50-E6.35 lock (distance up to ~8.5 Å), while antagonist amiloride keeps it stable (Figure 1 and Figure S3). Which are comparable to the ionic lock distance of the full agonist bound state structure of beta2 adrenergic receptor (9.97 Å) [27].

Figure S3 shows that in the agonist-bound state, one simulation replica exhibits the most pronounced ionic-lock disruption, the second replica shows transient breaking between 700–900 ns, and the third replica maintains the ionic lock throughout the entire 1 µs simulation. In contrast, in the antagonist-bound state, the ionic lock remains formed in all three replicas for the full duration of the simulations. In the apo state, the ionic lock remains formed in two replicas, while the third shows partial disruption. The differences among replicas likely reflect the stochastic nature of molecular dynamics simulations, in which local conformational rearrangements may occur at different times or may not be fully sampled within a single microsecond trajectory. Overall, the results support that agonist binding facilitates ionic-lock disruption, whereas antagonist binding stabilizes ionic-lock formation. The partial ionic-lock disruption observed in one apo replica is consistent with the presence of basal activity.

Our results show that S819 and amiloride use very different binding strategies in the TMD. S819 makes strong and stable contacts with TM3, TM5, and TM7, especially F3.40 (100% occupancy). In contrast, amiloride prefers TM4 (K4.43) and D7.32. The continuous S819-F3.40 contact is important because F3.40 is close to R3.50, and this may explain why the ionic lock breaks. Such “neighbor effects” are common in GPCR activation, where ligands disturb local residues near switches and cause larger conformational changes [31].

It is also notable that TM4 contributes little to S819 binding. Structural and modeling studies of the sweet receptor have shown that the main activation axis is TM3-TM5-TM7 [22,23]. Our data support this view and suggest that TM4 may only play a bigger role when the VFTD and CRD are present in the full receptor [18].

A key new finding is the salt bridge between D7.32 and R3.32. This interaction appears preferentially in the active state and shows opposite behavior to the classical ionic lock. In the antagonist system, amiloride occupies D7.32 and prevents it from forming this salt bridge. When S819 binds, D7.32 is released and forms a stable bridge with R3.32, helping to stabilize the active state.

This type of “partner switch,” where one salt bridge breaks and another interaction forms, has been described as a general rule in GPCR activation [31]. In Class C models of TAS1R2/TAS1R3, ligand binding was also shown to create new inter-helical interactions in the TMD [22]. The recent cryo-EM structure of the sweet receptor also showed that inter-helical contacts change significantly between apo and ligand-bound states [18]. Our data place D7.32 as a central hub that is blocked by antagonist but free to stabilize activation when agonist binds.

Our hydrogen bond analysis shows that activation of T1R2 involves a large reorganization of the network. Four main hydrogen bonds changed strongly between inactive and active states. For example, the Y2.56-D7.32 and T3.41-D5.47 bonds formed in the agonist condition, while the Y6.46-D5.47 and T1.54-S7.44 bonds were lost. This shows that residues change partners depending on the activation state.

Such hydrogen bond changes have also been reported in many GPCR studies, where different bonds appear in the inactive or active state to support the right conformation [31]. In Class C GPCRs, hydrogen bonds were suggested to work as important allosteric connections between helices [32]. Our findings show that the same idea is true for T1R2, with D7.32 again acting as a central switching residue.

Hydrophobic interactions also changed dramatically during activation. In the S819 state, new contacts formed between TM6, TM7, and TM2 (Y6.46-A7.42 and Y6.46-I7.43), while many TM5-TM6 and TM3-TM7 contacts present in the inactive state were lost. These changes indicate that TM6 shifts, releasing it from its inactive packing and allowing it to adopt an active orientation. This movement of TM6 is a well-conserved feature of GPCR activation [30,31]. Cryo-EM studies of Class C sweet receptors have also revealed hydrophobic rearrangements at the TM6-TM7 interface during activation [18]. Our simulations show how this process takes place in T1R2.

Our PCA and backbone angle analysis highlight TM7 intracellular flexibility, especially at the glycine residues G7.46 and G7.49. Glycines provide hinge-like flexibility and are often found at important turning points in GPCRs [30]. In the amiloride system, TM7 showed a π-helical structure, giving this region a more rigid shape. In the S819 system, the same TM7 segment changed into an α-helical structure, which is more flexible and can better support activation. This observation agrees with recent cryo-EM structures of TAS1R2/TAS1R3 that showed large rearrangements of TM7 between inactive and active states [18], supporting our finding that TM7 plays a central role in receptor activation.

Although our simulations focused on the T1R2 transmembrane domain (TMD) in the apo and ligand-bound forms generated by docking, no experimentally determined agonist-bound structure of the TMD is currently available. In this work, we specifically focused on the intrinsic activation properties of the T1R2 transmembrane domain, which represents the core structural region responsible for initiating receptor activation. It should be noted that published experimental evidence [14], as well as unpublished results from our laboratory, demonstrates that the hT1R2 or hT1R2-TMD monomer alone is functional. Future work incorporating the full heterodimer and experimentally resolved agonist-bound structures will be necessary to provide a more comprehensive understanding of receptor activation across all binding sites.

## 4. Materials and Methods

### 4.1. Molecular Docking

The hT1R2-TMD structure was obtained from recent cryo-EM structure (PDBID: 9NOR) [17], by extracting the TMD of T1R2 (residues 562–818). The hT1R2-TMD structure was prepared by the Protein Preparation Wizard module of Maestro (2024-3) program (Schrödinger, Inc., New York, NY, USA). The induced-fit-docking (IFD) simulations [26] were conducted for the sweetener S819 [15] and sweet inhibitor Amiloride to the receptor. The residues within 5 Å of ligand poses were selected for side chain optimization by prime refinement. The XP scores were used for ranking of the ligand poses, and the top 20 poses of docked ligand were saved for visual inspection and selection. The poses of the docked ligands with the best XP docking scores were selected as the predicted binding conformations.

### 4.2. MD Simulations

The predicted S819/Amiloride-hT1R2-TMD complexes and hT1R2-TMD (Apo), were used for MD simulations. Protonation states of the titratable residues in hT1R2-TMD receptor were calculated at pH = 7.4 using the H++ server (http://biophysics.cs.vt.edu/ (accessed on 14 September 2025)) [33]. The hT1R2-TMD or ligand-receptor complexes identified in the Induced Fit docking (IFD) were inserted into a simulated lipid bilayer composed of POPC–POPE–cholesterol (2:2:1) [34] and a water box using the CHARMM-GUI Membrane Builder webserver (http://www.charmm-gui.org (accessed on 14 September 2025)) [35]. Sodium chloride (150 mM) as well as neutralizing counter ions were applied to the systems. The total atom numbers are 88,668, 88,707, 88,691 for the hT1R2-TMD-APO, hT1R2-TMD-S819 and hT1R2-TMD-Amiloride, respectively. The PMEMD.CUDA program of AMBER 24 was used to conduct MD simulations [36]. The Amber ff19SB, lipid21 and TIP3P force field was used for the receptors, lipids, and water. The ligands, S819 and amiloride, were optimized using an ab initio quantum chemistry method at the HF/6-31G* level, followed by single-point energy calculations of the molecular electrostatic potential for charge fitting using Gaussian 16 (Gaussian, Inc., Wallingford, CT, USA) [37]. The restrained electrostatic potential charge-fitting scheme (RESP) was used to calculate partial charges on the atoms of the ligands [38]. The parameters of S819 and Amiloride were generated using general AMBER force field (GAFF) by the Antechamber module of AmberTools 25, using the partial charge determined via the RESP calculations. Coordinate files and system topology were established using the tleap module of Amber. The systems were energetically minimized by 500 steps (with a position restraint of 500 kcal/mol/Å2) followed by 2000 steps (without a position restraint) using the steepest descent algorithm. Heat was then applied to the systems to drive the temperature from 0 to 303 K using Langevin dynamics with a collision frequency of 1 ps−1. Receptor complexes were position-restrained using an initial constant force of 500 kcal/mol/Å2 during the heating process, which was subsequently diminished to 10 kcal/mol/Å2, allowing the lipid and water molecules free movement. Before the MD simulations, the systems underwent a 5 ns equilibration. Then, a total of 1000 ns of MD simulations were conducted using hydrogen mass repartitioning and a time step of 4 fs. The simulation temperature was set to 303 K (30 °C) to approximate near-physiological conditions while maintaining numerical stability during long MD simulations. At this temperature, the lipid bilayer remains in a fluid phase, preserving realistic membrane dynamics and protein–lipid interactions without excessive thermal fluctuations. The 303 K condition has been widely used in membrane protein simulations and provides a reliable balance between physiological relevance and computational stability. The coordinates were saved every 100 ps for analysis. The simulations were conducted in an isothermal and isobaric nature, with the pressure maintained using an isotropic position scaling algorithm with the pressure relaxation time fixed at 2 ps. Long-range electrostatics were calculated by the particle mesh Ewald method with a 10 Å cut-off [39]. Three replica simulations for each of the T1R2-TMD/Apo, T1R2-TMD/S819, and T1R2-TMD/Amiloride systems were performed. The results of the MD simulations were analyzed using various tools and methods, including the built-in utilities of the GROMACS program from Groningen University (Groningen, The Netherlands), Simulaid [29], as well as in-house scripts. For the residue labeling in the hT1R2 receptor, the classical (Ballesteros–Weinstein) numbering scheme [24] was employed.

### 4.3. Binding Free Energy Calculations

The MM-GBSA module, which is implemented in Amber24, was performed to calculate the binding free energy of S819 and Amiloride with the T1R2-TMD. The binding free energy can be decomposed into contributions from individual interacting residues of the channels, which consist of four energy terms: non-bonded electrostatic interaction, van der Waals energy in gas phase, polar, and non-polar solvation free energy. The polar component is calculated using a Generalized Born (GB) implicit solvation model. The non-polar component is calculated using a solvent accessible surface area model. 100 snapshots were extracted from the 900–1000 ns MD simulation trajectory at an interval of 1ns for the binding free energy calculations.

## 5. Conclusions

In this study, we used 1 μs MD simulations to compare the binding of agonist S819 and antagonist amiloride to the T1R2 transmembrane domain. Our results suggest that activation of T1R2 may depend on coordinated changes across several interaction networks. S819 appears to disrupt the classical R3.50-E6.35 ionic lock through persistent contact with F3.40, while amiloride maintains the lock in a stable state. We also observed a new salt bridge, D7.32-R3.32, which formed only in the active state. This finding highlights D7.32 as a potential molecular switch, blocked by antagonist but potentially stabilizing activation when agonist binds.

In addition to salt bridges, activation was associated with notable changes in hydrogen bond and hydrophobic networks. Four key hydrogen bonds appeared to switch partners between states, and hydrophobic contacts shifted from TM3-TM7/TM5-TM6 packing to a TM6-TM7-TM2 cluster, which may support the outward pivot of TM6. TM7 also transitioned from a more rigid π-helix to a flexible α-helix, with glycines G7.46 and G7.49 possibly acting as hinge points. Together, these observations are consistent with the idea that T1R2 activation follows general GPCR principles, while also involving Class C-specific structural features along a TM3-TM5-TM7 axis.

Overall, our study provides molecular insight into how sweet taste receptors may be activated and suggests D7.32 as a possible site for allosteric modulation. These findings could inform future efforts in rational drug or sweetener design.

## Data Availability

The original contributions presented in this study are included in the article/Supplementary Materials. Further inquiries can be directed to the corresponding author(s).

## Supplementary Materials

The following supporting information can be downloaded at: https://www.mdpi.com/article/10.3390/ijms262311464/s1.

## Abbreviations

STR	Sweet Taste Receptor
VFTD	Venus Flytrap Domain
GPCR	G protein-coupled receptor
CRD	Cysteine-Rich Domain
TMD	Transmembrane Domain
IFD	Induced Fit docking
PCA	Principal Component Analysis
DSSP	Dictionary of Secondary Structure in Proteins
cryo-EM	cryo-electron microscopy
