# Supplementary material for: Molecular Dynamics Insights into TAS1R2 Transmembrane Domain Activation
Source: Int J Mol Sci. 2025 Nov 26;26(23):11464. doi: 10.3390/ijms262311464 (PMC12692390; doi:10.3390/ijms262311464)
Supplement: Supplementary file 1 [file ijms-26-11464-s001.zip › Supplementary_T1R2_TMD_S819.pdf]

Supplementary Materials

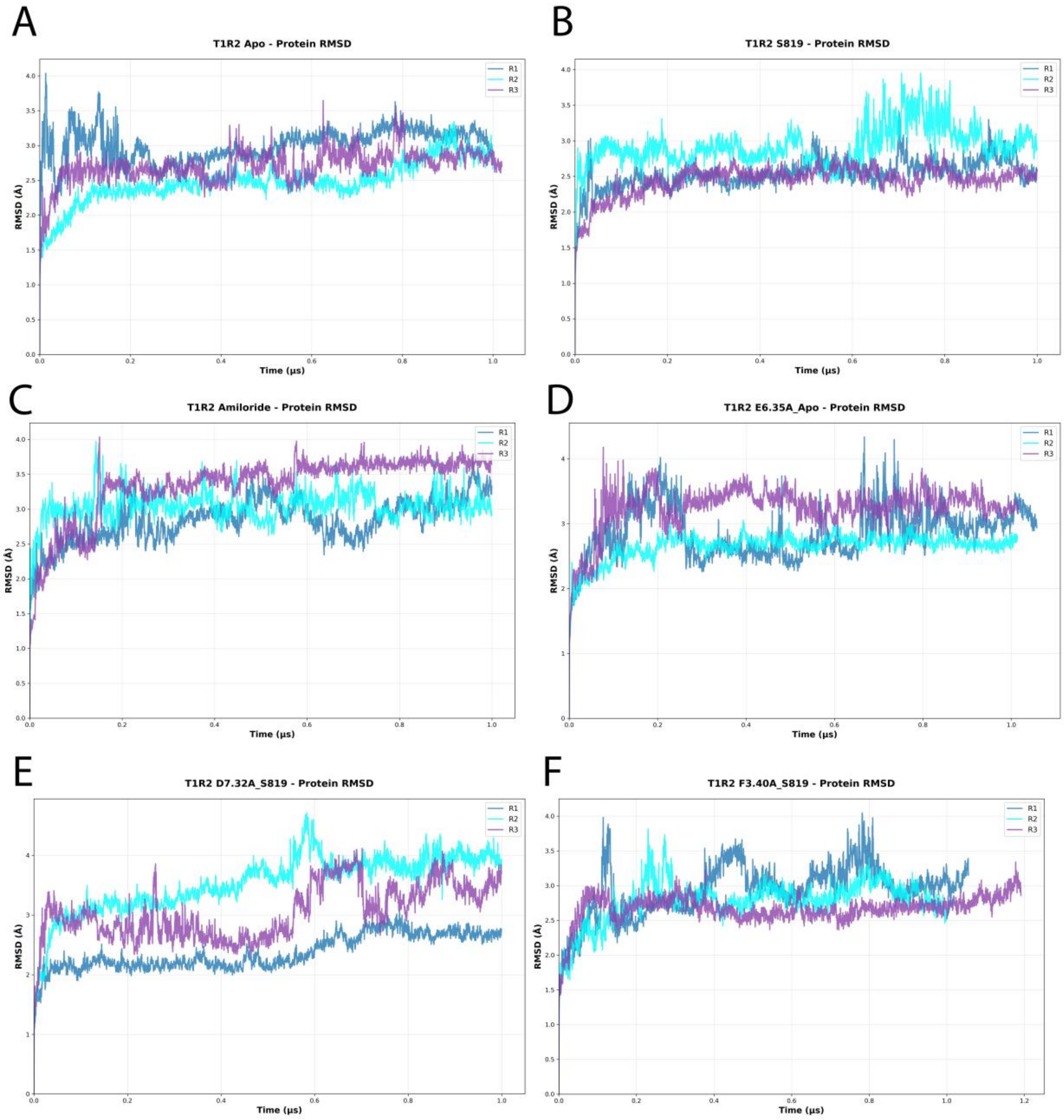

Figure S1. Root-mean-square deviation (RMSD) of the T1R2 transmembrane domain (TMD) during MD simulations. (A) Wild-type apo system. **(B)** Wild-type S819-bound system. **(C)** Wild-type amiloride-bound system. **(D)** Apo E6.35A mutant. **(E)** S819-bound D7.32A mutant. **(F)** S819-bound F3.40A mutant. Each panel shows the Cα RMSD (Å) as a function of simulation time (0-1 μs) for three independent replicas.

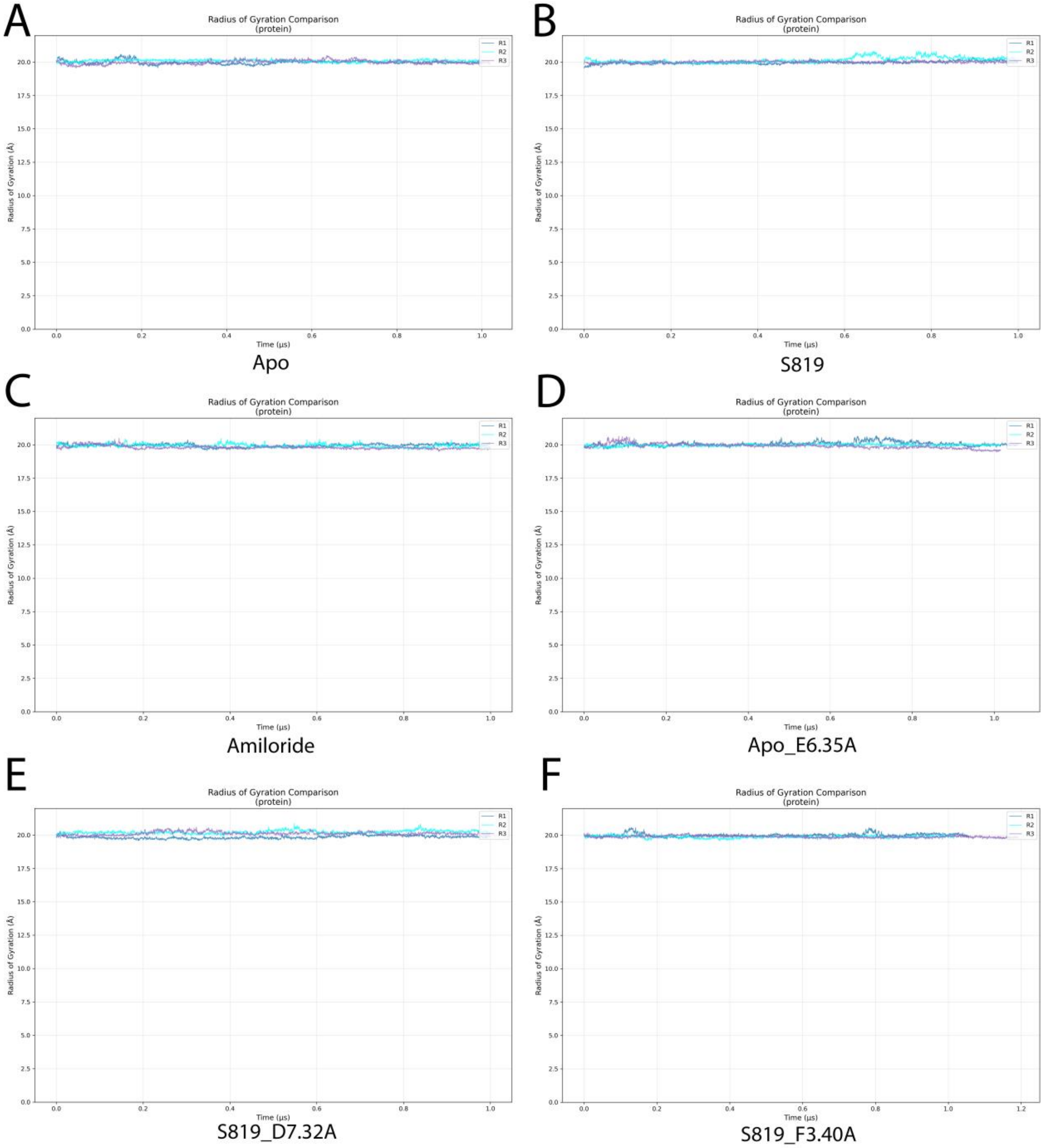

Figure S2. Radius of gyration (Rg) of the T1R2 transmembrane domain across systems and replicas. (A) Wild-type apo system. **(B)** Wild-type S819-bound system. **(C)** Wild-type amiloride-bound system. **(D)** Apo E6.35A mutant. **(E)** S819-bound D7.32A mutant. **(F)** S819-bound F3.40A mutant. Each panel shows the time-dependent Rg (Å) over three independent replicas during 1 μs simulations.

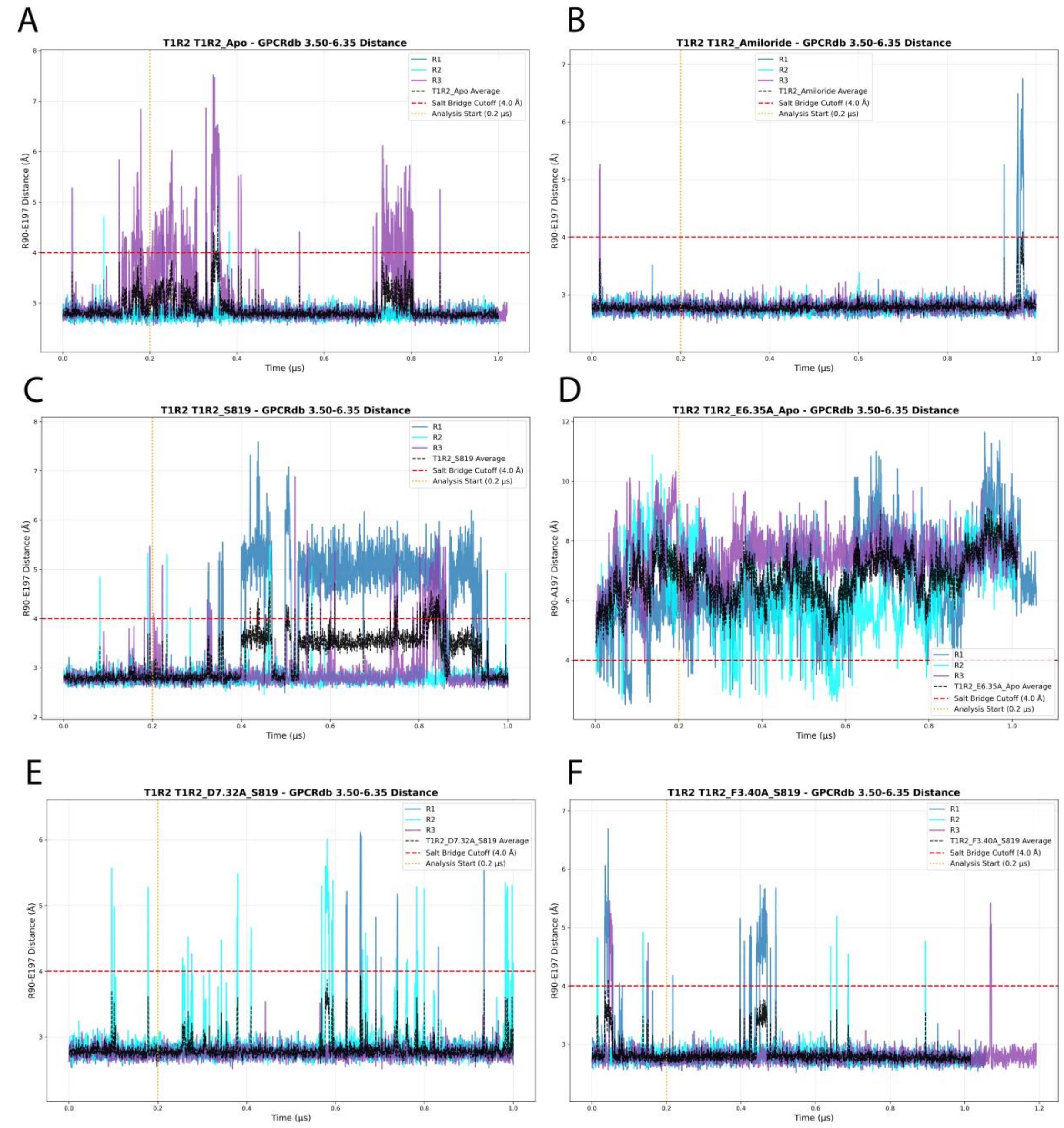

Figure S3. Time-dependent ionic lock (R3.50–E6.35) distance across systems and replicas. (A) Wild-type apo system. **(B)** Wild-type amiloride-bound system. **(C)** Wild-type S819-bound system. **(D)** Apo E6.35A mutant. **(E)** S819-bound D7.32A mutant. **(F)** S819-bound F3.40A mutant. Each panel shows the time evolution of the ionic lock distance over three independent replicas.

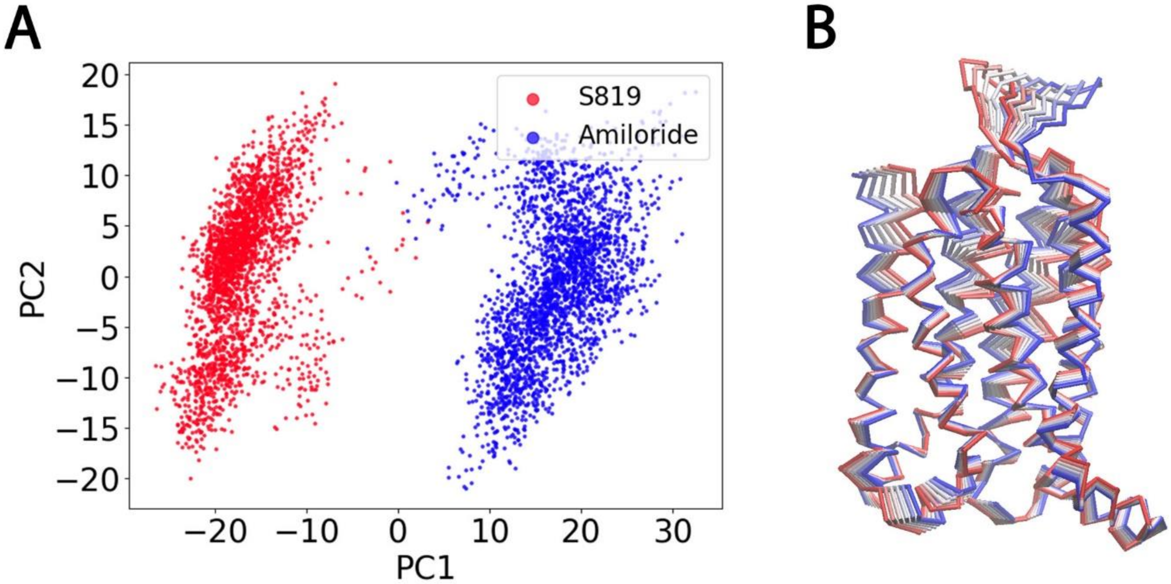

Figure S4. (A) Projection of the PCA results onto the first two principal components (PC1 and PC2) in 2D space. Red points represent the S819 system, and blue points represent the Amiloride system. **(B)** The first eigenvector (EV) from combined Principal Component Analysis (PCA) of T1R2-TMD/S819/Amiloride based on the MD simulations (100-1000 ns; Cα atoms of the receptor). The T1R2 receptor structures were shown as Cα traces (six frames colored from blue to red).

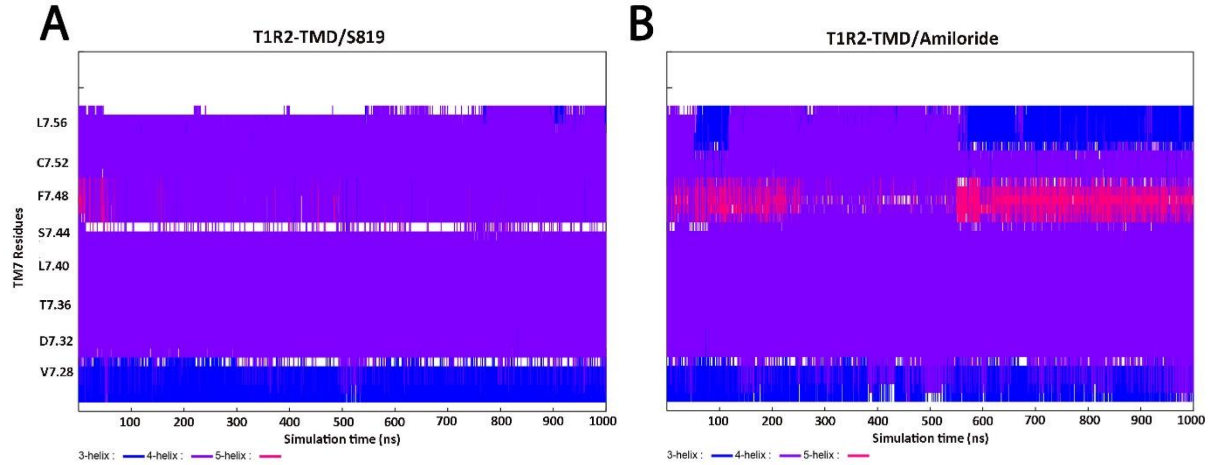

Figure S5. DSSP analysis results on the TM7 of T1R2-TMD by using Simulaid program.
(A) T1R2-TMD/S819. **(B)** T1R2-TMD/Amiloride.

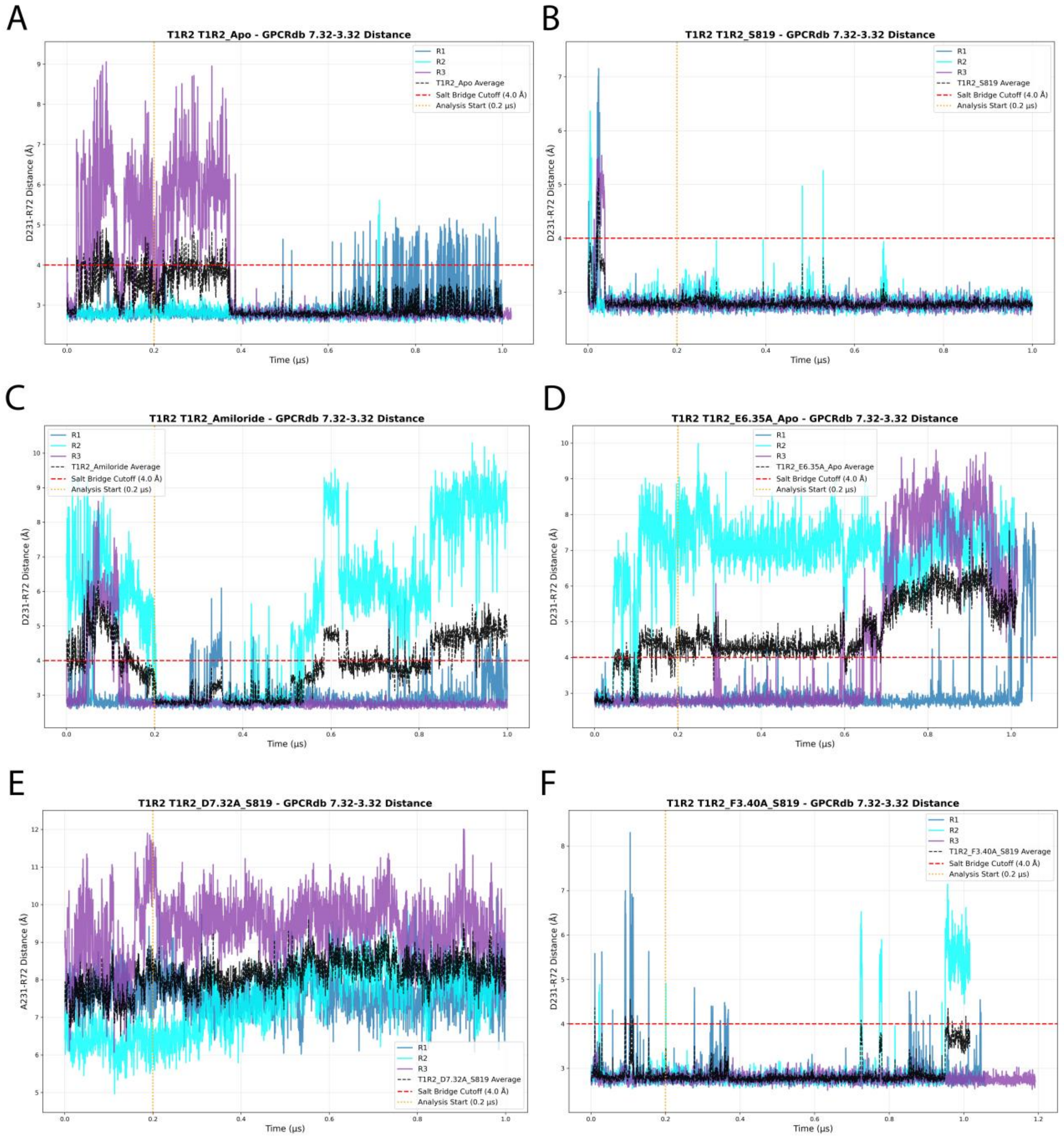

Figure S6. Time-dependent key salt bridge (D7.32–R3.32) distance across systems and replicas. (A) Wild-type apo system. **(B)** Wild-type S819-bound system. **(C)** Wild-type amiloride-bound system. **(D)** Apo E6.35A mutant. **(E)** S819-bound D7.32A mutant. **(F)** S819-bound F3.40A mutant. Each panel shows the time evolution of the key salt bridge distance over three independent replicas.

Table S1. Formed and broken Hydrogen Bond pairs in the T1R2/S819 system compared to the T1R2/Amiloride system from MD simulation trajectories (200 – 1000ns).

Formed			Broken		
Residue 1	Residue 2	Value	Residue 1	Residue 2	Value
D5.47x47	T3.41x41	1	Y6.46x46	D5.47x47	-0.63
S5.39x39	Q3.33x33	0.95	S7.44x45	T1.54x54	-0.75
N5.43x43	Q3.33x33	0.9			
Y6.46x46	S5.51x51	0.84			
K4.43x43	P3.37x37	0.83			
A7.42x43	S6.42x42	0.67			
D7.32x33	Y2.56x56	0.61			

Table S2. Formed and broken Hydrophobic interaction pairs in the T1R2/S819 system compared to the T1R2/Amiloride system from MD simulation trajectories (200 – 1000ns).

Formed			Broken		
Residue 1	Residue 2	Value	Residue 1	Residue 2	Value
A7.42x43	Y6.46x46	0.93	I7.30x31	V1.43x43	-0.6
L7.40x41	V2.53x53	0.91	V2.53x53	V1.43x43	-0.68
F2.40x40	W1.62x62	0.78	V2.53x53	A1.47x47	-0.75
I7.43x44	Y6.46x46	0.76	L7.33x34	Y2.56x56	-0.75
L7.45x46	I1.57x57	0.73	M6.43x43	A3.48x48	-0.76
F2.40x40	L1.58x58	0.69	L7.38x39	F6.45x45	-0.85
			Y6.46x46	L5.50x50	-0.87
			F6.47x47	V5.52x52	-0.88
			V7.37x38	F3.36x36	-0.89
			M6.57x57	L5.41x41	-0.93
			F6.47x47	L5.48x48	-0.94
			V6.51x51	L5.48x48	-0.99
			L7.33x34	F3.36x36	-1
			L7.40x41	F3.40x40	-1
